# Tie2 Expressing Monocytes in the Spleen of Patients with Primary Myelofibrosis

**DOI:** 10.1371/journal.pone.0156990

**Published:** 2016-06-09

**Authors:** Rita Campanelli, Gabriela Fois, Paolo Catarsi, Valentina Poletto, Laura Villani, Benedetta Gaia Erba, Luigi Maddaluno, Basilio Jemos, Silvia Salmoiraghi, Paola Guglielmelli, Vittorio Abbonante, Christian Andrea Di Buduo, Alessandra Balduini, Alessandra Iurlo, Giovanni Barosi, Vittorio Rosti, Margherita Massa, AGIMM Investigators

**Affiliations:** 1 Center for the Study of Myelofibrosis, Biotechnology Research Area, IRCCS Policlinico S. Matteo Foundation, Pavia, Italy; 2 Vascular Biology Group, IFOM, FIRC Institute of Molecular Oncology, Milano, Italy; 3 Unit of Hepatopancreatic Surgery, IRCCS Policlinico San Matteo Foundation, Pavia, Italy; 4 Hematology and Bone Marrow Transplant Unit, Azienda Ospedaliera Papa Giovanni XXIII, Bergamo, Italy; 5 Center for Research and Innovation for Myeloproliferative Neoplasms-CRIMM, AOU Careggi, and Department of Experimental and Clinical Medicine, University of Firenze, Firenze, Italy; 6 Biotechnology Research Area, IRCCS Policlinico S. Matteo Foundation, Pavia, Italy; 7 Department of Molecular Medicine, University of Pavia, Pavia, Italy; 8 Oncohematology Division, IRCCS Ca' Granda-Maggiore Policlinico Hospital Foundation, University of Milano, Milano, Italy; European Institute of Oncology, ITALY

## Abstract

Primary myelofibrosis (PMF) is a Philadelphia-negative (Ph−) myeloproliferative disorder, showing abnormal CD34^+^ progenitor cell trafficking, splenomegaly, marrow fibrosis leading to extensive extramedullary haematopoiesis, and abnormal neoangiogenesis in either the bone marrow or the spleen. Monocytes expressing the angiopoietin-2 receptor (Tie2) have been shown to support abnormal angiogenic processes in solid tumors through a paracrine action that takes place in proximity to the vessels. In this study we investigated the frequency of Tie2 expressing monocytes in the spleen tissue samples of patients with PMF, and healthy subjects (CTRLs), and evaluated their possible role in favouring spleen angiogenesis. We show by confocal microscopy that in the spleen tissue of patients with PMF, but not of CTRLs, the most of the CD14^+^ cells are Tie2^+^ and are close to vessels; by flow cytometry, we found that Tie2 expressing monocytes were Tie2^+^CD14^low^CD16^bright^CDL62^−^CCR2^−^ (TEMs) and their frequency was higher (p = 0.008) in spleen tissue-derived mononuclear cells (MNCs) of patients with PMF than in spleen tissue-derived MNCs from CTRLs undergoing splenectomy for abdominal trauma. By *in vitro* angiogenesis assay we evidenced that conditioned medium of immunomagnetically selected spleen tissue derived CD14^+^ cells of patients with PMF induced a denser tube like net than that of CTRLs; in addition, CD14^+^Tie2^+^ cells sorted from spleen tissue derived single cell suspension of patients with PMF show a higher expression of genes involved in angiogenesis than that found in CTRLs. Our results document the enrichment of Tie2^+^ monocytes expressing angiogenic genes in the spleen of patients with PMF, suggesting a role for these cells in starting/maintaining the pathological angiogenesis in this organ.

## Introduction

Primary myelofibrosis (PMF) is a Philadelphia-negative (Ph−) myeloproliferative disorder of unknown aetiology, characterized by the presence of acquired mutations of either JAK2, or MPL, or CALR genes in the myeloid cells of 90% of the patients [1], abnormal CD34^+^ progenitor cell trafficking [2], splenomegaly, and marrow fibrosis leading to extensive extramedullary haematopoiesis [3].

Neoangiogenesis has been documented in the bone marrow (BM) of patients with PMF, and microvessel density has been reported to be higher than in other chronic myeloproliferative disorders [4]. Similarly, it has been shown an up to three-times increased capillary vascular density in the spleen of patients with PMF and splenomegaly, suggesting a significant contribution of neoangiogenesis to the spleen volume expansion [5]. This hypothesis is strengthened by previous observations of reduction of spleen volume with anti-angiogenic therapy [6, 7], and by the observation that responders to the treatment were patients with a documented increase in BM angiogenesis [8]. In keeping with this notion we documented an increased number of circulating putative endothelial progenitor cells (EPCs) in the peripheral blood (PB) and spleen of patients with PMF [9], and an increased frequency of endothelial colony forming cells (ECFCs) in the PB of patients with PMF and with myeloproliferative disorders with high risk of splanchnic vein thrombosis [10].

CD14^low^CD16^bright^ non classical monocytes expressing the angiopoietin-2 receptor (Tie2) have been shown to support abnormal angiogenic processes in solid tumors through a paracrine action that takes place in close proximity to the vessels [11, 12, 13]. More recently, CD14^bright^CD16^low^ intermediate monocytes have also been reported to selectively up-regulate the expression of genes codifying for Tie2, the vascular endothelial growth factor (VEGF) receptor 2, and endoglin, and have been defined as angiogenic monocytes [14]. In this line of evidence, in patients with PMF we reported an increased frequency of PB CD14^bright^CD16^low^Tie2^+^ angiogenic monocytes that was correlated with the severity of the disease [15].

In this study we have investigated the frequency of CD14^low^CD16^bright^ non classical monocytes expressing Tie2 in spleen tissue samples of patients with PMF and healthy subjects undergoing splenectomy for clinical reasons or abdominal trauma, respectively. To the best of our knowledge, no data are available on the presence of angiogenic monocytes in the spleen of patients with PMF, and their possible role in fuelling the well documented abnormal angiogenesis that characterizes this organ.

## Materials and Methods

### Patients

Sixteen patients with PMF in whom splenectomy was indicated because of refractory and symptomatic splenomegaly, entered the study (Table 1). The diagnosis of PMF was established according to World Health Organization Criteria [16]. At time of sampling patients were receiving androgens (n = 3), oncocarbide (n = 8), steroids (n = 2; one until 6 months before splenectomy), or red cell transfusions (n = 1); two patients were out of therapy (withdraw ruxolitinib 6 and 3 months before splenectomy, respectively). A risk class was assigned to all the patients included in the study according to the DIPSS score [17]. Spleen tissue samples were obtained from 6 CTRLs who underwent splenectomy for traumatic lesions (median age 50, range 32–65).

**Table 1:** Characteristics of patients with primary myelofibrosis (PMF) whose spleen was available for this study.

	n	
**Age, years, median (range)**	16	65 (51–78)
**Males, number (%)**		(54.5%)
**Spleen size, (cm**^**2**^**), median (range)**	8	467 (300–744)
**Time from diagnosis to splenectomy, months, median (range)**	16	67 (13–202)
**CD34**^**+**^ **cells in peripheral blood/microliter, median (range)**	16	68 (19–429)
Dupriez risk score*, number (%)		
**0**		4 (25)
**1**		7 (44)
**2**		5 (31)

* A DIPSS score of 0 (low risk) was assigned for haemoglobin level greater than 10 g/dl and a blood cell count between 4x10^9^/l and 30x10^9^/l, score of 1 (intermediate risk) for either a haemoglobin level less than 10g/dl or a white blood cell count greater than 30x10^9^/l or less than 4x10^9^/l, and a score of 2 (high risk) if both the haemoglobin level and white blood cell count were in the aberrant ranges.

PB of 73 consecutive patients with PMF, collected from 14 Italian Centers participating to the Italian Registry of MF, were studied (S1 Table). Patients (95%) were studied before receiving treatment; the spleen size and index [18], prognostic score and severity score [19] were calculated. PB samples from 13 healthy individuals (CTRLs) 7 males and 6 females (median age 53 years, 23–68) were studied.

The study was approved by the IRCCS Policlinico S. Matteo Foundation’s institutional review board (approval number 2011–0004143). All clinical investigations have been conducted according to the principles expressed in the Declaration of Helsinki. Written informed consent was obtained from each patient included in the study.

### Spleen tissue

Spleen tissue specimens from 3 patients with PMF and 3 CTRLs were embedded in OCT compound (VWR, Leuven, Belgium), snap-frozen in liquid nitrogen and cut into 3-μm-thick sections. Alternatively, tissue specimens were reduced to a single cell suspension by Gentle MACS Dissociator (Miltenyi Biotec GmbH, Bergisch Gladbach, Germany) and mononuclear cells (MNCs) obtained by density gradient centrifugation. CD3^+^ and CD14^+^ cells were purified from spleen tissue-derived MNCs by immunomagnetic selection (MACS, Miltenyi Biotec). Megakaryocytes (MKs) were obtained *in vitro* from CD34^+^ spleen tissue-derived cells and cultured as previously reported [20].

Spleen tissue derived MNCs, CD14^+^ monocytes, or CD3^+^ cells and MKs were cultured in 24-well plate (2x10^6^/well) in RPMI 1640 medium supplemented with 10% FCS (both Euroclone SpA, Milan, Italy). After 3 days at 37°C, 5% CO_2_, the supernatants were collected, centrifuged at 1200 rpm for 10 min and the conditioned medium (CM) stored at -80°C.

### Flow cytometric analysis of Tie2-expressing monocytes

**Fig 1:**
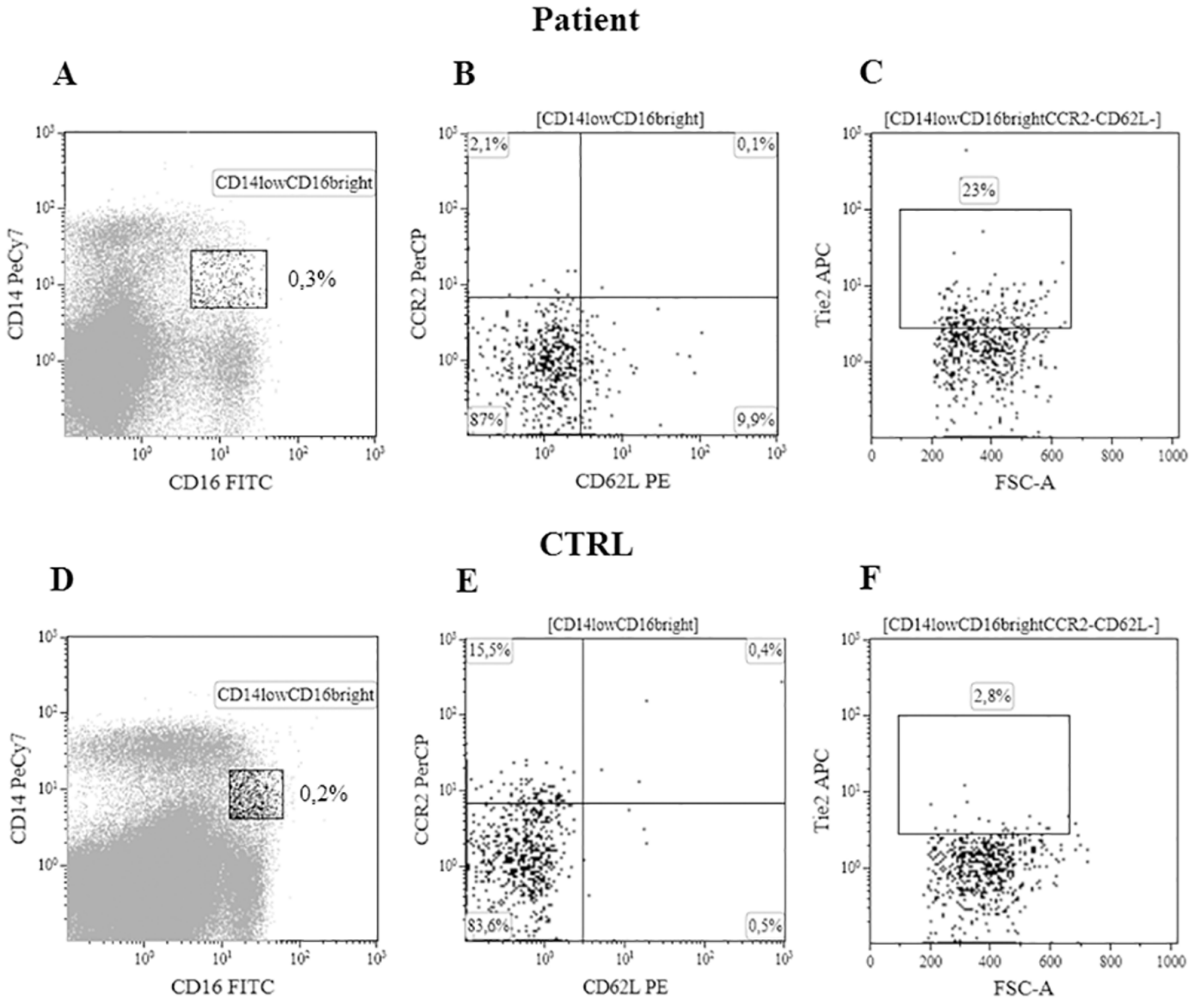
Cytofluorimetric analysis of spleen tissue derived TEMs. CD14^low^CD16^bright^ non classical monocytes from a patient with PMF were gated (panel A); CD14^low^CD16^bright^ not expressing the CD62L and CCR2 antigens on the membrane (panel B) were than evaluated for the expression of Tie2 (panel C). Panels D, E, F for one representative healthy subject (CTRL).

Spleen tissue-derived or PB MNCs were stained with PeCy7-anti-CD14, FITC-anti-CD16, APC-anti-Tie2, PE-anti-CD62L and PerCP-anti-CCR2 (R&D Systems, Minneapolis, MN or eBioscience, San Diego, CA). According to Venneri et al. [13] angiogenic monocytes, acquired by a flow cytometer (Navios^™^; Beckman Coulter, Inc, Brea, CA) and analyzed by Kaluza^®^ flow analysis software (Beckman Coulter), were identified as Tie2^+^ CD14^low^CD16^bright^CD62L^−^CCR2^−^ cells. The appropriate isotype controls were used to set the markers for positive/negative cells (see Fig 1 and S1 Fig). We evaluated the percentage of total CD14^+^ cells in PB or spleen tissue-derived MNCs of patients with PMF and CTRLs; the percentage of Tie2^+^ cells was investigated on MNCs, CD14^+^, CD14^low^CD16^bright^, and CD14^low^CD16^bright^CD62L^−^CCR2^−^ cells. Data were shown as median (range).

### Immunofluorescence and confocal microscopy

Spleen tissue samples snap-frozen in liquid nitrogen were cut into 3-μm-thick sections. Following fixation, permeabilization, and blocking, sections were incubated with rabbit anti-human Tie2 (polyclonal) and mouse anti-human CD14 (monoclonal) antibodies. Alexa Fluor 488 anti-rabbit, and Alexa Fluor 555 anti-mouse (Invitrogen, Carlsbad, CA) were used as secondary antibodies. Slides were counterstained with DAPI (Sigma Aldrich, St. Luis, MO). The images, obtained by confocal laser scanning microscopy (Leica TCS SP2, 63x objective), were processed by Photoshop software (Adobe Systems Incorporated, Mountain View, CA, USA).

The frequency of CD14^+^ and CD14^+^Tie2^+^ cells in the spleen tissue sections of patients with PMF and CTRLs were calculated by ImageJ software (National Institute of Health, USA, http://rsbweb.nih.gov/ij/.) using the formula: number of CD14^+^Tie2^+^ x 1000/total DAPI^+^ cells in each section.

### In vitro angiogenesis assay

Endothelial colony forming cells (ECFCs) were obtained from PB of healthy subjects, as previously described [10]. Cells from early passage (P1–P2) ECFCs were isolated by trypsinization and resuspended in EBM2 medium (Lonza, Basel CH) in the presence of 10% FCS. Two x 10^4^ ECFC-derived cells per well were plated onto 96 well plates coated with Cultrex^®^ basement membrane extract (Trevigen^®^, Gaithersburg, MD, USA), in the presence of 20% of CM from spleen derived MNCs, immunomagnatically selected CD3^+^ or CD14^+^ cells, or MKs. Plates were then incubated at 37°C, 5% CO_2_ and capillary-like network formation was assessed starting from 4 hours later and up to 24 hours. At least 2 different sets of cultures were performed for each experimental point. Quantitative evaluation of the total length of tube-like structures was assessed by measuring the ratio of the tube length in the assays performed with MNC, MNC subset, or MK CM (mm)/surface of the field (mm^2^) by ImageJ software (National Institute of Health, USA, http://rsbweb.nih.gov/ij/.).

### Sorting of Tie2^+^ monocytes

CD14^+^ cells of 2 patients with PMF were selected from spleen tissue-derived MNCs by immuno-magnetic selection (MACS, Miltenyi Biotec). From pooled CD14^+^ cells, CD14^+^Tie2^+^ and CD14^+^Tie2^−^ cells were then sorted by FACS-sorting (BD FACSAria^™^ II, BD Biosciences) with purity greater than 95% for each subset (S2 Fig).

### RNA isolation and whole-transcriptome amplification (WTA)

RNA from CD14^+^, CD14^+^ Tie2^+^, and CD14^+^ Tie2^−^ sorted cells was purified by RNeasy Plus Micro kit (Qiagen, Hilden, Germany) and used for amplification (50 ng for the CD14^+^ cells and the total RNA sample for the CD14^+^ Tie2^+^ and CD14^+^ Tie2^−^ cells). Library preparation was performed following the distributor’s (Sigma-Aldrich, St Louis, MO, USA) recommendations for the Complete Whole Transcriptome Amplification Kit (S1 Appendix).

### Gene expression analysis

Predesigned primers (Sigma-Aldrich) were used for EvaGreen assays (S2 Table). Quantification of transcripts was carried out in a 15 μl reaction mix containing 1X SsoFast EvaGreen Supermix (Bio-Rad, Hercules, CA, USA), 400 nM of each primer and 10 ng of purified WTA-cDNAs. The PCR data were collected using the CFX96 Real-Time System (Bio-Rad). Each sample was tested in duplicate. Calculation of normalized relative expression levels (fold change) and error propagation (standard error) was done using the Qbase Plus software version 3 (Biogazelle, Gent, Belgium). Normalization was performed using the two most stably expressed reference genes which were selected using the geNorm algorithm, among the following candidates: YWHAZ, GAPDH, HPRT1, UBC, B2M. A validation of results was performed (S1 Appendix).

### Statistical analysis

Groups were compared by means of Mann-Whitney *U*-test for unpaired samples, one-way-repeated-measures-ANOVA and Bonferroni’s test as a post hoc test, or Wilcoxon for paired samples. All computations were performed with STATISTICA software (StatSoft, Inc.Tulsa, OK).

## Results

### Patient characteristics

The clinical characteristics of patients with PMF enrolled at time of splenectomy (n = 16) of whom we obtained spleen tissue samples are shown in Table 1 (see Material and Methods section).

### Perivascular CD14^+^Tie2^+^ cells in the spleen tissue samples of patients with PMF

**Fig 2:**
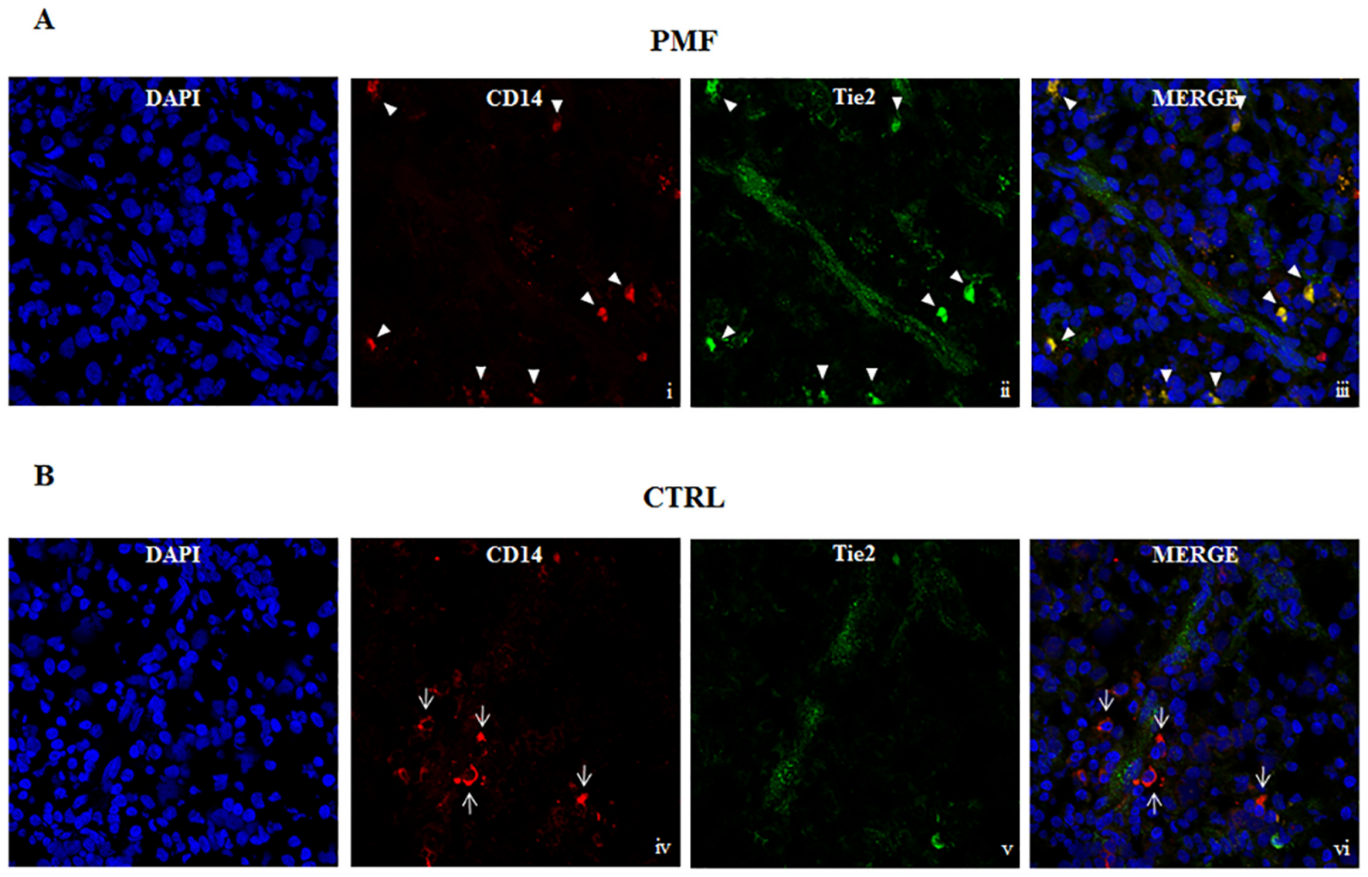
Confocal microscopy images of spleen tissue samples stained with anti-CD14 and anti-Tie2. Spleen tissue samples were immunostained for the indicated markers and a small capillary from each stained specimen was chosen for display. A) Arrow heads indicate cells expressing both CD14 in red (i) and Tie2 in green (ii) in the perivascular area of spleen tissue sample from 1 representative patient with primary myelofibrosis (PMF). The yellow colour indicates colocalization of CD14 and Tie2 (iii). B) Thin arrows indicate CD14^+^ cells (iv) not expressing Tie2 (v, vi) in spleen tissue sample from 1 representative CTRL. DAPI (blue) was used as the nuclear marker and the images were incorporated in the merge panels (iii, vi). Images were obtained at 63x magnification.

We performed immunofluorescence studies on snap frozen spleen tissue sections of patients with PMF (n = 3) and CTRLs (n = 3). Our staining protocol did not include the anti-CD16 antibody since it is well known that only CD16^+^ monocytes can express the Tie2 antigen [13]. We found that in the spleen tissue samples from patients with PMF the most of the CD14^+^ cells co-expressed Tie2 on the membrane and some of them were close to vessels (Fig 2, upper panels for one representative patient). In the 3 spleen tissue sections from patients with PMF the median frequency of CD14^+^ cells was 44/1000 cells (range 41–48), while the median frequency of CD14^+^Tie2^+^ cells was 20/1000 cells (range 14–25). In spleen tissue sections from CTRLs (n = 3), the median frequency of CD14^+^ cells was comparable (median 30/1000, range 9–52) to that found in tissue sections from patients with PMF; however, in none of the sections were observed monocytes co-expressing the Tie2 antigen (Fig 2, lower panels for one representative CTRL).

### Evaluation of Tie2^+^ monocytes in the spleen tissue-derived MNCs of patients with PMF and CTRLs

To further investigate the phenotype of the CD14^+^Tie2^+^ cells evidenced by confocal microscopy, by flow cytometry we evaluated the total CD14^+^ or Tie2^+^ cells on spleen tissue derived MNCs of patients with PMF (n = 16) and healthy subjects (CTRLs) (n = 6). In addition, we measured the percentage of Tie2^+^ cells on CD14^+^, CD14^low^CD16^bright^ non classical monocytes, and CD14^low^CD16^bright^CD62L^−^CCR2^−^ cells. As shown in Table 2, while the percentage of CD14^+^ cells or Tie2^+^ cells among the MNCs was comparable between patients and CTRLs, the percentage of CD14^+^ cells expressing Tie2 was higher (p = 0.027) in patients with PMF than in CTRLs. Similarly, the frequency of CD14^low^CD16^bright^ monocytes expressing Tie2 was significantly higher (p = 0.028) in the spleen tissue-derived MNCs of patients with PMF than in CTRLs. We focalized on this last cell subset assessing the lack of membrane antigens such as CD62L (a member of the selectin family of adhesion molecules that facilitate rolling of inflammatory leukocytes along the vascular endothelium) and CCR2 (the monocyte-chemoattractant protein-1 receptor, a chemokine involved in the recruitment of inflammatory monocytes), defining monocytes recruited through non inflammatory pathways (i.e. angiogenic pathways). The percentage of CD14^low^CD16^bright^CD62L^−^CCR2^−^ cells expressing Tie2 (TEMs) [13] was significantly higher (p = 0.008) in the spleen tissue-derived MNCs of patients with PMF than of CTRLs (Table 2, Fig 1 and S1 Fig for the cytofluorimetric analysis of one representative patient and CTRL).

**Table 2:** Evaluation of CD14^+^ cell and cell subsets in spleen derived MNCs of patients with primary myelofibrosis (PMF) and of healthy subjects (CTRLs). Data are shown as median (range).

		PMF	CTRLs	
	Evaluated as	(n = 16)	(n = 6)	p vs CTRLs
CD14^+^	% MNCs	7.3(0.7–25)	7.0(2.0–19)	0.6
Tie2^+^	% MNCs	6.0(1.6–41.3)	2.3(0.4–24)	0.13
Tie2^+^	%CD14^+^	27(6.0–70)	6.4(1.8–16)	0.027
Tie2^+^	% CD14^low^CD16^bright^	8.0(2.3–50)	3.0(0.2–5.4)	0.028
Tie2^+^	% CD14^low^CD16^bright^CD62L^−^CCR2^−^(TEMs)	9.2(0.8–47.5)	1.6(0–6.8)	0.008

We evaluated TEMs in paired samples of PB MNCs (blood sample was obtained just before the splenectomy) and spleen tissue-derived MNCs of patients with PMF (n = 6). The percentage of TEMs was significantly higher (p = 0.040) in the spleen compartment than in the PB; in addition, the PB TEM frequency was significantly lower (p = 0.002) in patients with PMF than in CTRLs (n = 13) who volunteered a PB sample (Table 3).

**Table 3:** Evaluation of TEMs in paired samples of peripheral blood (PB) and spleen tissue derived MNCs of patients with primary myelofibrosis (PMF) and in the PB of healthy not splenectomised subjects (CTRLs). TEMs are shown as median (range).

	n	PB TEMs	Spleen TEMs	p
**PMF**	6	4.8(0–11)	27 (2.6–55.6)	0.04
**CTRLs**	13	20.2 (10–29)	NA	NA
**p PMF vs CTRLs**		0.002		

### Spleen recruitment of TEMs in patients with PMF

To evaluate the hypothesis of a recruitment of TEMs from the PB to the spleen compartment we analysed PB TEMs in 73 consecutive patients with PMF; we divided these patients according with their spleen size at time of sampling. Patients with a spleen size within the range of CTRLs (≤100 cm^2^) had a frequency of PB TEMs significantly higher (p = 0.000003) than that found in patients with a spleen size greater than 100 cm^2^ (range 108–782 cm^2^), and comparable to that of CTRLs (Table 4).

**Table 4:** Evaluation of TEMs in the peripheral blood (PB) of patients with primary myelofibrosis (PMF) divided according with the spleen size. Data are shown as median (range).

Patients with PMF	n	PB TEMs	pvs CTRLs
**Spleen size ≤ 100 cm**^**2**^	35	24.1 (2.2–34.2)*	0.07
**Spleen size >100 cm**^**2**^ (range 108–782 cm^2^)	38	8.1 (0–38)	0.002

* p = 0.000003 patients with spleen size ≤100 cm^2^ vs patients with spleen size >100 cm^2^

To further investigate this aspect, we evaluated the frequency of PB TEMs of patients with PMF (n = 9) assessed after splenectomy, with a median time from the surgical intervention of 30 months (range 2–96). In these patients the percentage of circulating CD14^+^ cells (12, range 5–27) was comparable to that of CTRLs; the percentage of PB TEMs (27.5, range 2–57) was comparable to that of CTRLs and significantly higher (p = 0.02) than that of patients with PMF tested before splenectomy (Table 3).

### Increased angiogenic potential of spleen tissue derived MNC conditioned medium (CM) from patients with PMF

**Fig 3:**
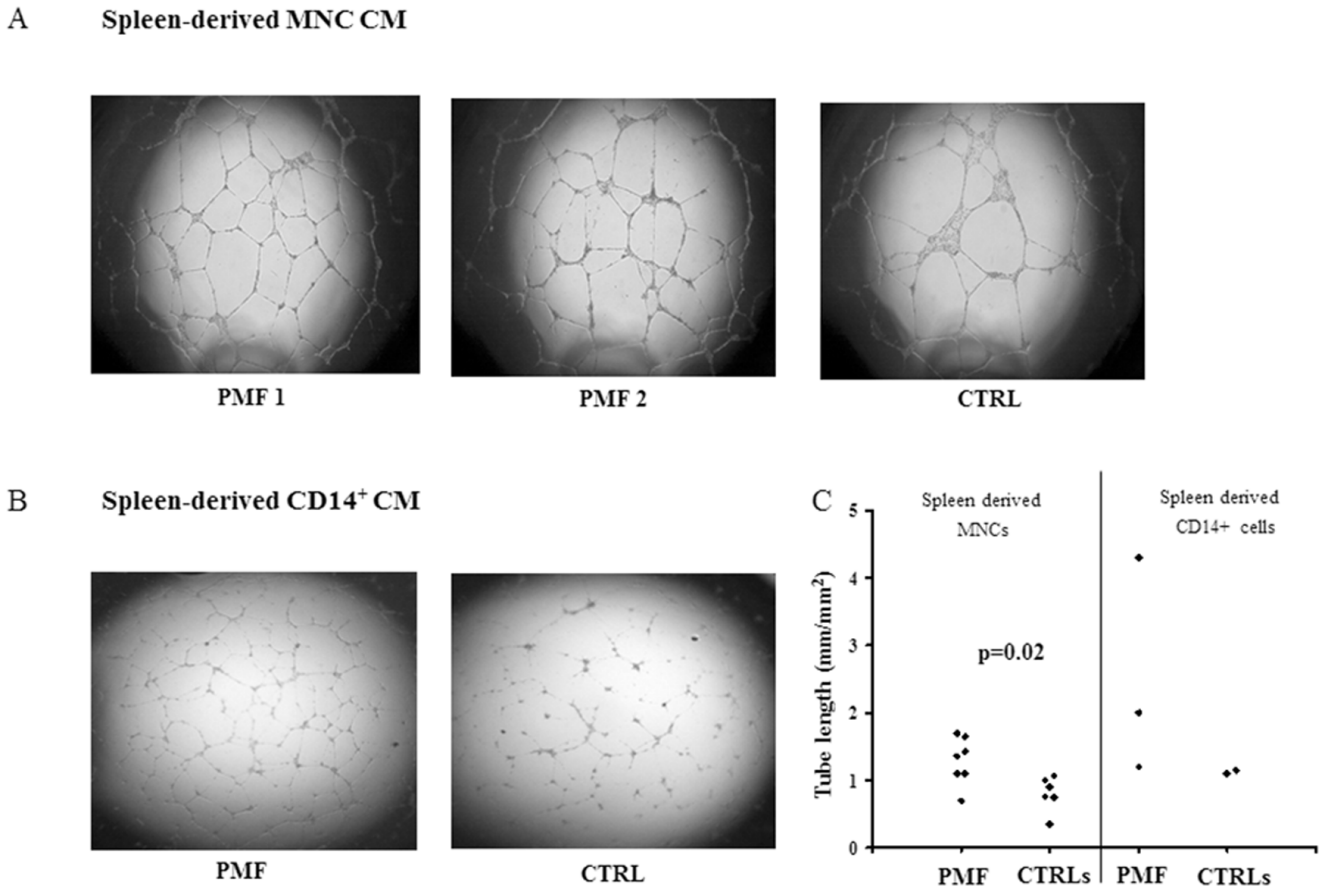
*In vitro* tubulogenesis induced by spleen MNC or selected CD14^+^ cell CM. Digital images of endothelial tubes obtained by bright-field light microscopy 24 hours after plating Endothelial Colony Forming Cells from a healthy donor on Matrigel-coated wells in presence of CM of spleen tissue derived MNCs from two patients with primary myelofibrosis (PMF) and one representative CTRL (panel A), or CM of spleen tissue derived selected CD14^+^ cells from one representative patient with PMF and one CTRL (panel B). Cultures were examined under an inverted microscope (Labovert, Leitz, Germany) in bright field, at 2.5X magnification using a PL (Leitz Wetzlar, Germany) objective; quantitative evaluation of the tube-like structures, expressed as ratio of the total length of tubular structures per field (mm)/surface of the field (mm^2^) (ImageJ software by National Institutes of Health, USA, http://rsbweb.nih.gov/ij/.), of all the patients with PMF and CTRLs tested is shown (panel C).

We cultured spleen derived MNCs in RPMI 1640 medium supplemented with 10% FCS for 3 days at 37°C (see Material and Methods section); the CM samples of 7 patients with PMF and 6 CTRLs were added to ECFC-derived ECs from a healthy subject to evaluate their *in vitro* capacity to induce tube-like structure formation. We observed that the net of tube-like structure was denser in presence of the spleen MNC CM of patients with PMF than in presence of the spleen MNC CM of CTRLs (Fig 3, panel A for 2 representative patients and 1 CTRL). The formation of tube-like structures with unconditioned medium was performed and resulted negligible (not shown). The total length of tube-like structures, assessed by measuring the ratio of the tube length in the assays performed with spleen MNC CM (mm)/surface of the field (mm^2^), was significantly higher (p = 0.02) in presence of the CM from patients with PMF with respect to that obtained with CM from CTRLs (Fig 3, panel C, left side).

To better characterize, among the spleen derived MNCs, those producing angiogenic factors, we separately cultured MKs (see Material and Methods section), and immuno-magnetically selected CD3^+^ or CD14^+^ cells from patients with PMF (n = 3) and CTRLs (n = 2). When CMs from these cultures were added to ECFC-derived ECs from a healthy subject, we observed that the total length of tube-like structures was increased in presence of the CM from CD14^+^ cells of 2 out of 3 patients with PMF with respect to that obtained with CMs from CD14^+^ cells of CTRLs (Fig 3, panel B for a representative patient and a CTRL). The formation of tube-like structures with CMs from CD3^+^ cells or MKs was negligible (not shown). The quantitative evaluation of the tube-like structures obtained with the CD14^+^ CM of either patients with PMF and CTRLs is shown (Fig 3, panel C, right side).

### Gene signature of spleen tissue derived CD14^+^Tie2^+^ cells

In order to investigate the pro-angiogenic activity of monocytes, we immuno-magnetically purified total CD14^+^ cells from 2 patients with PMF; from pooled CD14^+^ cells CD14^+^Tie2^+^, and CD14^+^Tie2^−^ cells were sorted (S2 Fig). Purification of CD14^+^Tie2^+^ cells was technically challenging because of their low frequency; we therefore used a quantitative PCR approach that allows to analyse gene expression from small amounts of mRNA (see Material and Method section). To study the nature of CD14^+^Tie2^+^ cells, we measured the expression of genes previously implicated in angiogenesis, tissue remodelling, immune response, cell adhesion, chemotaxis, and haematopoiesis (S2 Table). A list of the genes differentially expressed in CD14^+^Tie2^+^ with respect to CD14^+^Tie2^−^ cells and total CD14^+^ cells is shown (Table 5). Among them there were genes codifying monocyte/macrophage-derived cytokines *(IL1β*), and matrix related proteins such as Heparanase (*HPSE*) that have been widely demonstrated to increase microvessel density and reduce survival of cancer patients. In addition, we evidenced a high mRNA expression of genes that promote angiogenesis and cell survival, thymidine phosphorylase/endothelial cell growth factor 1 (*TYMP/Ecgf1*) and Catenin beta1 (*CTNNB1*), important mediators of epithelial cell-cell adhesion, further supporting the pro-angiogenic role of these cells. Similarly, the higher expression of the gene codifying for CXCR4 on monocytes has been described to characterize populations of monocytes with angiogenic and reparative potential.

**Table 5:** Genes differentially expressed in CD14^+^Tie2^+^ and CD14^+^Tie2^−^ or total CD14^+^ cells (CD14^+^ tot).

Genes	Fold change (SEM)				
	CD14^*+*^Tie2^+^vs CD14^*+*^Tie2^−^	CD14^*+*^Tie2^+^vs CD14^*+*^ tot	ΔCq Tie2^+^	ΔCq Tie2^−^	ΔCq CD14^+^ tot
***CXCR4***	11.7 (0.6)	0.6 (0.1)	2.7	5.4	1.2
***CTNNB1***	6.9 (0.6)	3.0 (0.3)	4.1	6.1	4.8
***TIMP1***	3.1 (0.2)	1.4 (0.1)	6.2	7.0	5.9
***SPI1***	4.1 (0.2)	0.9 (0.1)	8.4	9.6	7.3
***TYMP***	n.a.	36.5 (1.3)	3.1	n.a.	7.5
***HPSE***	18.6 (1.8)	2.1 (0.2)	7.3	10.7	7.5
***IL1B***	5.3 (0.5)	1.2 (0.1)	9.2	10.8	8.5

The expression level of each gene in Tie2^+^ is indicated as fold-change versus Tie2^−^ or CD14^+^ tot. The values were calculated using the two most stable reference genes (*UBC* and *GAPDH*).

ΔCq is used to represent the expression level of each gene compared to the others in each cell type (approximately the lower the ΔCq the higher the expression level). The ΔCq of each gene was calculated using *UBC* as reference gene.

Each data point represents the average of 2 technical replicates of the same sample.

## Discussion

In this study we assessed the frequency of TEMs in the spleen of patients with PMF, their possible origin, and their role in fuelling the spleen abnormal angiogenesis.

In fact, we have previously shown by immuno-histochemistry that, although the frequency of circulating EPCs was increased, no strict correlation was ever found between the degree of spleen angiogenesis and these cells evaluated by either cytofluorimetric analysis or *in vitro* culture of ECFCs [9 and personal observations].

In the last years, a number of studies performed in animal models have been published attributing to Tie2 expressing monocytes a role in favouring angiogenesis through a paracrine action [11, 21, 22]. The results in humans are more discordant. Matsubara et al described CD14^low^CD16^bright^Tie2^+^ angiogenic monocytes both in the PB and tumor site of patients with hepatocellular carcinoma; these cells were increased and correlated with the therapeutic response, the degree of tumoral vascularisation, and were observed in close proximity to vessels in tumor tissue [23]. At variance, Goede et al studied patients with colorectal cancer and found no correlation among the frequency of CD14^+^Tie2^+^ cells in both PB and tumor tissue, the tumor stage or the angiogenic processes [24]. These discrepancies may be related to the different kind of tumors studied and the different phenotypic analysis of these cells; however, the contribution of Tie2^+^ monocytes belonging to the non classical/intermediate subset to the angiogenic processes has been recently confirmed in a study that describes their role in improving the revascularization of the ischemic limb in both animal models and humans [21].

In a previous study our group documented the significant increase of the angiogenic subset of CD14^bright^CD16^low^ intermediate monocytes expressing Tie2^+^ in the PB of patients with PMF, and the possible use of this parameter as a biomarker [15].

In this study, by confocal microscopy, we showed that CD14^+^ cells co-expressing Tie2 accumulate in spleen tissue of patients with PMF, some in close proximity to vessels, without showing direct connections, while no monocyte expressing Tie2 is present in spleen tissue samples from CTRLs. These novel findings were all in agreement with data found in human and murine solid tumors, where monocytes were described to contribute to the tumor angiogenesis [11, 13].

Interestingly, when we analysed the spleen tissue derived angiogenic monocytes from patients with PMF by flow cytometry, the highly represented phenotype was Tie2^+^CD14^low^CD16^bright^ CD62L^−^CCR2^−^ (TEMs) at variance with our previous finding in the PB compartment, where the CD14^bright^CD16^low^ intermediate monocytes expressing Tie2 were significantly increased [15]. These data suggest that angiogenic monocytes migrating toward tissues are non classical monocytes (i.e. macrophages), while circulating angiogenic monocytes belong to an earlier maturation step (intermediate monocytes) [14].

It has recently been described a treatment-related increase of CD14^bright^CD16^low^ intermediate monocytes due to glucorticoids [25]; however, since only 1/16 patients with PMF was receiving steroids at time of splenectomy, while the most was receiving oncocarbide that induce mainly myelosuppression [26], our data indicate that the increased frequency of TEMs in the spleen of patients with PMF can be considered disease-related.

Regarding the origin of TEMs found in the spleen tissue, it is important to point out that the spleen of patients with PMF is one of the organs where extra-medullary haematopoiesis is active after BM fibrosis takes place. In addition, we found that the frequency of TEMs is higher in spleens with a size greater than 100 cm^2^ with respect to those with a size minor than 100 cm^2^; therefore, the generation of the monocyte/macrophage lineage, possibly including angiogenic monocytes, within the organ could be hypothesized. However, supporting the recruitment of angiogenic monocytes from the PB, their frequency in spleen tissue-derived MNCs was higher than in paired samples of PB from patients with PMF. Moreover, in patients with PMF who underwent splenectomy (median of 30 months from the surgery) there was a significant increase of circulating angiogenic monocytes, with a PB frequency comparable to that of CTRLs. Nevertheless, we cannot exclude that after the splenectomy the (re)activation of new spots of haematopoiesis either in the BM or in other extra-medullary sites, such as liver or lungs, could be responsible of the increased number of circulating angiogenic monocytes, while the apparent recruitment may be related to other organs (i.e. BM).

Regarding the possible role of angiogenic monocytes in fuelling the spleen abnormal angiogenesis, it has been described that they promote angiogenesis through a paracrine action in close proximity to vessels. This possibility has been shown by seeding sorted CD14^+^Tie2^+^ or CD14^+^Tie2^−^ cells in presence of endothelial cells (i.e. Huvec) [22]. Two major limitations did not allow us to perform similar experiments: the difficulty in collecting spleen tissue samples since the splenectomy is becoming a rare, infrequent indication after the jak2 inhibitors (i.e. Ruxolitinib) have become available in clinical practice. In addition, the low number of spleen-derived CD14^+^Tie2^+^ cells obtained by sorting, not sufficient to assess their role in co-culture experiments. The low efficiency of the sorting procedure could be due to the long lasting disease and the abnormal spleen size (i.e.10 times the normal one) that characterize the patients included in this study; the number of angiogenic monocytes in the organ may not be as high as in the early phase of the “angiogenic switch”, therefore our results may reflect what is left of the initial phenomenon. Nevertheless, this study documents the presence of angiogenic factors in the CM of spleen derived MNCs of patients with PMF resulting in the formation of an abnormal capillary network, denser than that measured with spleen derived MNC CM from CTRLs; more importantly, CD14^+^ cells appear to be the MNC subset responsible of the production of the angiogenic factors.

In an attempt to investigate the expression of genes involved in the angiogenic activity of monocytes, we evaluated the gene expression profile of sorted CD14^+^Tie2^+^, CD14^+^Tie2^−^ and CD14^+^ cells pooled from the spleen samples of 2 patients with PMF. Although the number of genes that we were able to validate by our technical approach is low (S2 Table) some of the transcripts over expressed in CD14^+^Tie2^+^ cells (*IL1β*, *HPSE*, *TYMP*) have been previously reported among the genes that characterize the expression profile of solid tumor TEMs, belonging to those required for tumor angiogenesis [27]. In fact, IL1β is a major cytokine linking inflammation and angiogenesis in tumor neoangiogenesis [28] and it has been reported to cause the rapid expression of *TYMP*, an important enzyme involved in the nucleoside metabolism, that is induced by inflammation or micro-environmental stress, in particular as a consequence of chemotherapeutic treatment [29, 30]. Its over expression protects cells from apoptosis, promotes angiogenesis and it has been found in a wide range of human cancer tissues [31]. Similarly, HPSE, CTNNB1 and TIMP-1 over expression has been described to be implicated in both pathological and physiological processes focusing on angiogenesis [32–34] as well as a significant high expression of CXCR4 has been reported to characterize monocytes with presumed reparative/angiogenic properties [35]. The transcription factor SPI1 (PU.1), whose link with the V617F Jak2 mutation has been described [36], has also been associated, in a chick yolk-sac experimental model system, with an angiogenic activity of macrophages that is mediated by the RXR family of nuclear receptors [37]. Thus, our molecular investigations bring support to a possible role played by Tie2^+^ monocytes in favouring neoangiogenesis in the spleen of patients with PMF.

In conclusion our data suggest that angiogenic monocytes accumulate in the spleen of patients with PMF in close proximity of vessels, and that this phenomenon likely modulates the peripheral trafficking of these cells. They also show that spleen derived angiogenic monocytes from patients with PMF express genes involved in angiogenesis that may contribute to the formation of an abnormal capillary network.

## Supporting Information

S1 Appendix RNA isolation and whole transcriptome amplification (WTA). (DOC)

S1 Fig Cytofluorimetric analysis of spleen tissue derived TEMs. CD14^low^CD16^bright^ non classical monocytes from a patient with PMF were gated (panel A); isotype controls (IgG1 PE and IgG2b PerCP) of gated CD14^low^CD16^bright^ (panel B); CD14^low^CD16^bright^ not expressing the CD62L and CCR2 antigens on the membrane (panel C, lower left quadrant); isotype control (IgG1 APC) of CD14^low^CD16^bright^CD62L^−^CCR2^−^ cells (panel D); CD14^low^CD16^brigh^tCD62L^−^CCR2^−^ cells expressing Tie2 (panel E). Panels F, G, H, I and L show the same analysis in spleen derived MNCs from a healthy subject (CTRL).
(TIF)

S2 Fig Gating strategy during the sorting of Tie2 expressing monocytes. Representative flow cytometric dot plot of CD14^+^ cells immune-magnetically purified from spleen tissue-derived mononuclear cells of 2 pooled patients with PMF (A) co-stained with anti-Tie2 monoclonal antibody (B). CD14^+^Tie2^+^ cells (B, top right gate C) and CD14^+^Tie2^−^ cells (B, low right gate D) have been sorted by BD FACSAria^™^ II instrument.
(TIF)

S1 Table Clinical characteristics of patients with primary myelofibrosis (PMF) who did not undergo splenectomy. (DOC)

S2 Table Predesigned primers for EvaGreen Assays. (XLSX)
