# Supplementary material for: Tie2 Expressing Monocytes in the Spleen of Patients with Primary Myelofibrosis
Source: PLoS One. 2016 Jun 9;11(6):e0156990. doi: 10.1371/journal.pone.0156990 (PMC4900622; doi:10.1371/journal.pone.0156990)
Supplement: S1 Appendix — (DOC) [file pone.0156990.s001.doc]

**S1 supplementary appendix**

*RNA isolation and whole-transcriptome amplification (WTA)*

CD14^+^ mononuclear cells were isolated from a patient’s spleen by density gradient centrifugation and subsequent immunomagnetic selection (Miltenyi Biotec). CD14^+^Tie2^+^ and CD14^+^Tie2^-^ cells were isolated from these total CD14^+^ cells by cell sorting with the method described above. RNA from single samples was purified with the RNeasy Plus Micro kit (Qiagen), using the gDNA eliminator spin column before the RNeasy Minelute spin column for RNA purification. Twenty µl of a RNase-free water solution of dithiothreitol 2 M (Bio-rad) was added to 1 ml of RLT-plus lysis buffer, before homogenizing the cells. Finally, the total RNA was eluted with 14 µl of RNase-free water. Different proportions of the RNA samples were used for amplification according to the measured or expected RNA content: 50 ng of purified RNA for the CD14^+^ cells and the total RNA sample for the CD14^+^Tie2^+^ and CD14^+^Tie2^-^ cells.

Library preparation was performed following the distributor’s (Sigma-Aldrich, St Louis, MO, USA) recommendations for the Complete Whole Transcriptome Amplification Kit (catalog no. WTA2). SYBR Green (Sigma-Aldrich) was added to the amplification reaction, which was performed in a CFX96 real-time instrument (Bio-Rad) to monitor yield. When the SYBR Green signal reached a plateau, the reaction was stopped. Amplified cDNA was purified using a spin column protocol with a cutoff of 100 bp (Macherey-Nagel, Duren, Germany) and quantified by measuring 260 nm absorbance. Nucleic acids were quantified with a Nanodrop 1000 spectrophotometer (ThermoScientific).

*Gene expression analysis*

Samples for gene expression analysis were obtained as described above. Predesigned primers (Sigma- Aldrich; catalog no. KSPQ12012) were used for EvaGreen assays (Table S2). Quantification of transcripts was carried out in a 15 µl reaction mix containing 1X SsoFast EvaGreen Supermix (Bio- Rad) and 400 nM of each primer. The PCR conditions were 95°C for 30 sec followed by 45 cycles of 95°C for 5 sec and 58°C for 5 sec. Melting curves were generated after amplification in the range 65–95°C with increments of 0.2°C every 10 sec. For each experiment, 4 µl of purified cDNA (corresponding to 10 ng of cDNA) were used. The PCR data were collected using the CFX96 Real- Time System (Bio-Rad). Each sample was tested in duplicate. Calculation of normalized relative expression levels (fold change) and error propagation (standard error) was done using the Qbase Plus software version 3 (Biogazelle). Normalization was performed using the two most stably expressed reference genes which were selected using the geNorm algorithm, among the following candidates: YWHAZ, GAPDH, HPRT1, UBC, B2M (S2 Table). A validation procedure of the results, consisting of three steps, was applied. In the first step, the assays were performed on three samples where RNA extraction and reverse transcription were performed according to a standard method (Catarsi et al. Plos One 2015). An aliquot of these three samples containing 50 ng of RNA was amplified with the method of Whole Transcriptome Amplification described above, and the obtained cDNAs were also used for the qPCR. The results obtained by amplifying the two types of cDNA were compared and the valid assays have been identified (132/148; 89%). The second step consisted in selecting assays who had given amplification in CD14^+^ cells and at least one sample of the couple CD14^+^ / CD14^+^Tie2^+^ or CD14^+^/ CD14^+^ Tie2^-^ (47/132; 35.6%). The last step of validation was performed by selecting only the assays in which the ratio between the standard error and fold change was less than 0.2 in amplified samples (7/47; 15%).
