# Supplementary figures and images for: Tie2 Expressing Monocytes in the Spleen of Patients with Primary Myelofibrosis
Source: PLoS One. 2016 Jun 9;11(6):e0156990. doi: 10.1371/journal.pone.0156990 (PMC4900622; doi:10.1371/journal.pone.0156990)

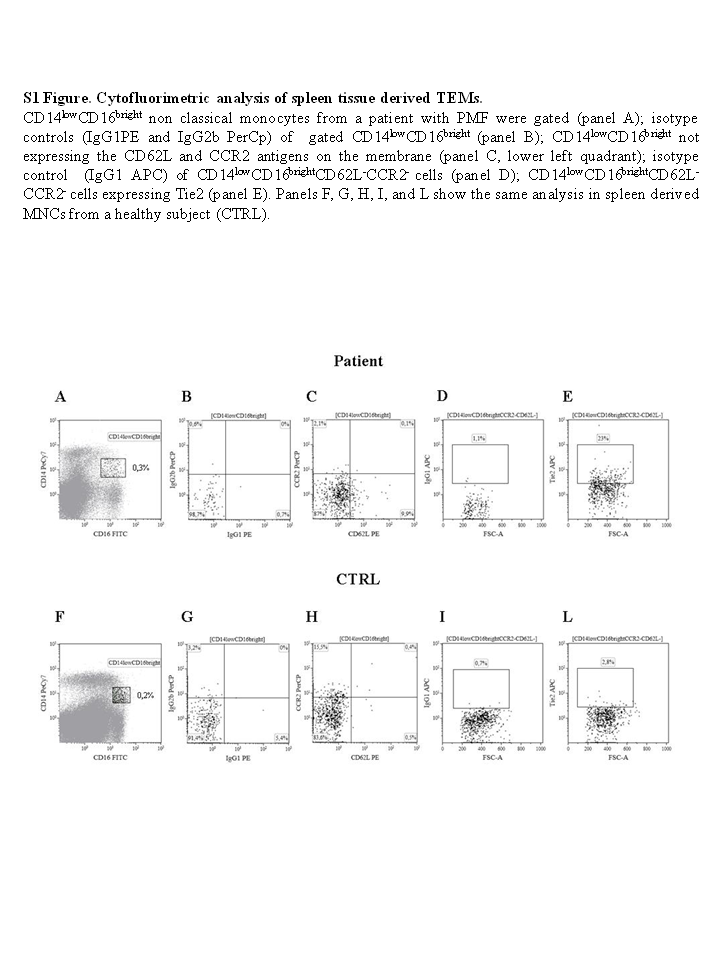

Supplement: S1 Fig — CD14lowCD16bright non classical monocytes from a patient with PMF were gated (panel A); isotype controls (IgG1 PE and IgG2b PerCP) of gated CD14lowCD16bright (panel B); CD14lowCD16bright not expressing the CD62L and CCR2 antigens on the membrane (panel C, lower left quadrant); isotype control (IgG1 APC) of CD14lowCD16brightCD62L−CCR2− cells (panel D); CD14lowCD16brightCD62L−CCR2− cells expressing Tie2 (panel E). Panels F, G, H, I and L show the same analysis in spleen derived MNCs from a healthy subject (CTRL). (TIF) [file pone.0156990.s002.tif]

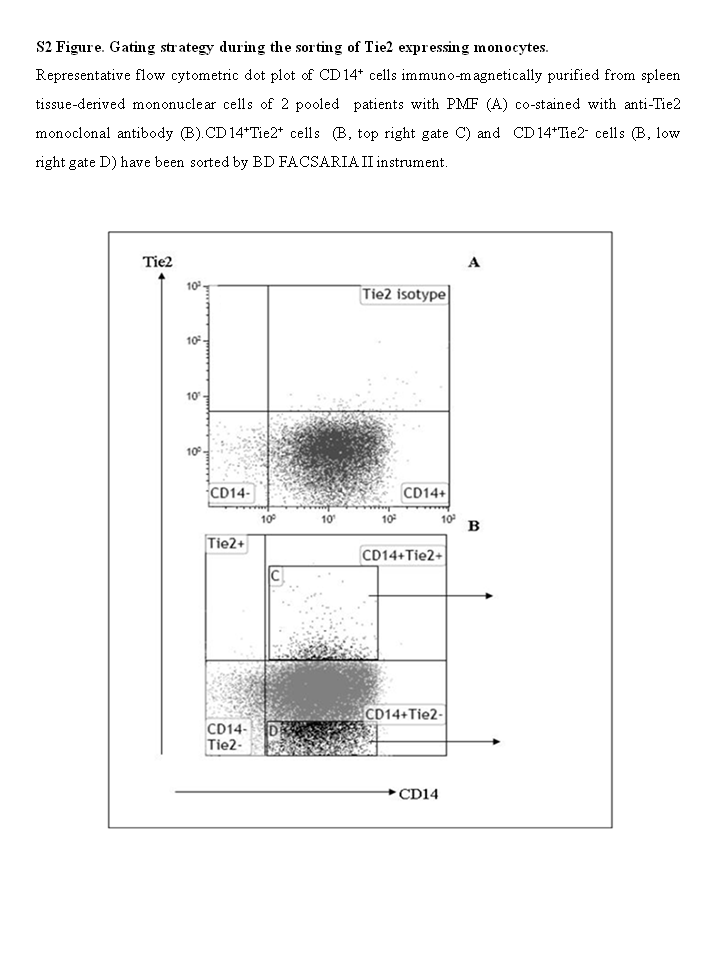

Supplement: S2 Fig — Representative flow cytometric dot plot of CD14+ cells immune-magnetically purified from spleen tissue-derived mononuclear cells of 2 pooled patients with PMF (A) co-stained with anti-Tie2 monoclonal antibody (B). CD14+Tie2+ cells (B, top right gate C) and CD14+Tie2− cells (B, low right gate D) have been sorted by BD FACSAria™ II instrument. (TIF) [file pone.0156990.s003.tif]
