# Supplementary material for: Tie2 Expressing Monocytes in the Spleen of Patients with Primary Myelofibrosis
Source: PLoS One. 2016 Jun 9;11(6):e0156990. doi: 10.1371/journal.pone.0156990 (PMC4900622; doi:10.1371/journal.pone.0156990)
Supplement: S1 Table — (DOC) [file pone.0156990.s004.doc]

**S1 Table.** Clinical characteristics of patients with primary myelofibrosis (PMF) who did not undergo splenectomy.

|  | No. patients | Median  (range) |
| --- | --- | --- |
| **Age, years, median (range)** | 73 | 52 (22-80) |
| **Males** | 48 |  |
| **Hemoglobin concentration (g/L)** | 73 | 125 (65.4-165) |
| **<80** | 3 | 79 (65.4-79) |
| **>80 <100** | 18 | 91 (80.5-99.1) |
| **>100 <120** | 16 | 112 (102-119) |
| **>120** | 36 | 138 (122-165) |
| **White blood cell count x109/L** | 73 | 8 (1.8-27.8) |
| **<4** | 7 | 3 (1.8-3.98) |
| **>4 <15** | 58 | 7.8 (4.3-27.2) |
| **>15 <30** | 8 | 20.6(16-27.2) |
| **>30** | 1 | 44 |
| **Platelet count x109/L** | 53 | 391 (8.3-1503) |
| **<40** | 1 | 8.3 |
| **>40 <150** | 15 | 110(44-143) |
| **>150 <600** | 37 | 330(164-575) |
| **Time from diagnosis to examination (months)** |  | 32.5(0-341) |
| **Dupriez score*, number**  **0**  **1**  **2** | 50  18  5 |  |
| **Severity score^** |  |  |
| **<3** | 43 |  |
| **>3** | 17 |  |

* A DIPSS score of 0 (low risk) was assigned for haemoglobin level greater than 10 g/dl and a blood cell count between 4x10^9^/l and 30x10^9^/l, score of 1 (intermediate risk) for either a haemoglobin level less than 10g/dl or a white blood cell count greater than 30x10^9^/l or less than 4x10^9^/l, and a score of 2 (high risk) if both the haemoglobin level and white blood cell count were in the aberrant ranges.

^ The severity score is the sum of severity points derived from the values of haemoglobin concentration, white blood cell count, platelet count, and spleen volume.
